# Supplementary material for: Extreme synchronization transitions
Source: Nat Commun. 2025 May 15;16:4505. doi: 10.1038/s41467-025-59729-8 (PMC12081737; doi:10.1038/s41467-025-59729-8)
Supplement: Supplementary file 1 — Supplementary Information [file 41467_2025_59729_MOESM1_ESM.pdf]

Supplementary Information

accompanying the manuscript

Extreme Synchronization Transitions

Seungjae Lee,[1,*] Lennart J. Kuklinski,[1] and Marc Timme[1,2,3,4,†]

[1] *Chair for Network Dynamics, Institute of Theoretical Physics and Center for Advancing Electronics Dresden (cfaed), Technische Universität Dresden, 01062 Dresden, Germany*

[2] *Cluster of Excellence Physics of Life, Technische Universität Dresden, 01062 Dresden, Germany*

[3] *Center Synergy of Systems, Technische Universität Dresden, 01062 Dresden, Germany*

[4] *Lakeside Labs, Lakeside B04b, 9020 Klagenfurt, Austria*

(Dated: April 22, 2025)

SYSTEM STATES AND ASYMPTOTICS

In the main article, we discuss a complex locked state for a purely imaginary coupling ($\beta = 0$) and for its asymptotics as $\beta \to 0^+$. Here, we explain the details of the analysis and provide additional numerical support.

A system of complexified Kuramoto oscillators $z_\mu = x_\mu + \mathrm{i}y_\mu \in \mathbb{C}$ is governed by [1, 2]

$$\frac{\mathrm{d}}{\mathrm{d}t} z_\mu = f_\mu(\boldsymbol{z}) := \omega_\mu + \frac{K}{N}\sum_{\nu=1}^{N} \sin(z_\nu - z_\mu) \tag{S.1}$$

for $\mu \in [N] := \{1, 2, \cdots, N\}$ where $\boldsymbol{z} := (z_1, \cdots, z_N)^\top \in \mathbb{C}^N$. Here, the natural frequency of each unit is a constant $\omega_\mu \in \mathbb{R}$ with a constraint $\sum_{\mu=1}^{N} \omega_\mu = 0$ and we complexified a coupling constant $K = |K|e^{\mathrm{i}\alpha} \in \mathbb{C}$ to be a complex constant. For $\beta = 0$ ($\alpha = \frac{\pi}{2}$; a purely imaginary coupling), the system (S.1) reads

$$\frac{\mathrm{d}}{\mathrm{d}t} x_\mu = \omega_\mu - \frac{|K|}{N}\sum_{\nu=1}^{N} \cos(x_\nu - x_\mu)\sinh(y_\nu - y_\mu) \tag{S.2}$$

$$\frac{\mathrm{d}}{\mathrm{d}t} y_\mu = \frac{|K|}{N}\sum_{\nu=1}^{N} \sin(x_\nu - x_\mu)\cosh(y_\nu - y_\mu) \tag{S.3}$$

for $\mu \in [N]$. A complex locked state, i.e., a fixed point solution, is achieved by setting (S.3) to zero, leading to $x_\mu^{(0)} = 0$ for all $\mu \in [N]$. Substituting this into (S.2), we obtain an algebraic equation

$$\frac{\omega_\mu}{|K|} = \frac{1}{N}\sum_{\nu=1}^{N} \sinh(y_\nu^{(0)} - y_\mu^{(0)}) \tag{S.4}$$

for $\mu \in [N]$. Assuming that $y_\mu^{(0)} = -\sinh^{-1}(b\tilde{\omega}_\mu)$ where $\tilde{\omega}_\mu := \frac{\omega_\mu}{|K|}$ (Fig. S1), the parameter b is determined by

$$\tilde{\omega}_\mu = \frac{1}{N}\sum_{\nu=1}^{N} \sinh y_\nu^{(0)} \cosh y_\mu^{(0)} - \frac{1}{N}\sum_{\nu=1}^{N} \cosh y_\nu^{(0)} \sinh y_\mu^{(0)} = b\tilde{\omega}_\mu \frac{1}{N}\sum_{\nu=1}^{N} \sqrt{1 + (b\tilde{\omega}_\nu)^2} \tag{S.5}$$

$$\Longrightarrow \frac{1}{b} = \frac{1}{N}\sum_{\nu=1}^{N} \sqrt{1 + (b\tilde{\omega}_\nu)^2}. \tag{S.6}$$

The first term in (S.5) cancels out due to the constraint $\sum_{\mu=1}^{N} \omega_\mu = 0$. Hence, the complex locked state for a purely imaginary coupling reads

$$z_\mu^{(0)} = x_\mu^{(0)} + \mathrm{i}y_\mu^{(0)} = 0 - \mathrm{i}\sinh^{-1}(b\tilde{\omega}_\mu) \tag{S.7}$$

for $\mu \in [N]$.

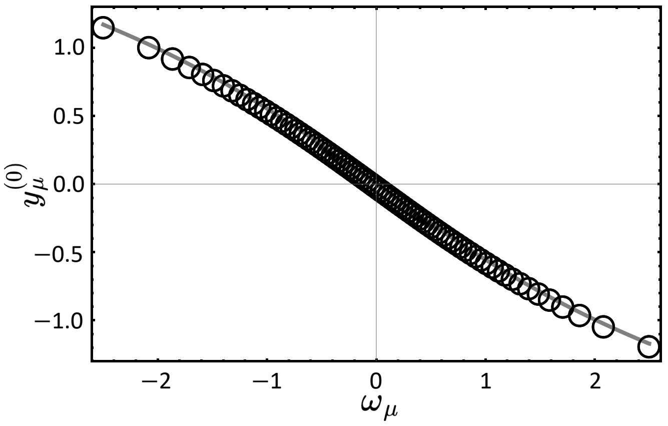

FIG. S1. **Complex locked state for a purely imaginary coupling.** Imaginary parts ($y_\mu^{(0)}$) of a complex locked state are depicted as a function of natural frequencies (ω_μ) for $\beta = 0$ (i.e., $\alpha = \frac{\pi}{2}$), $|K| = 1.5$ and $N = 80$ (see the main text). The black open circles indicate numerically observed data, whereas the gray solid curve indicates $y(\omega) = -\sinh^{-1}(\frac{b\omega}{|K|})$.

It is noteworthy that the parameter b has the properties listed below. First, it follows from (S.6) that $b > 0$ for any set of natural frequencies and for any $|K| > 0$. Second, in the thermodynamic limit $N \to \infty$, we obtain a self-consistency equation:

$$1 = b_\infty \int_{-\infty}^{\infty} \sqrt{1 + \frac{b^2\omega^2}{|K|^2}} g(\omega) \mathrm{d}\omega \Longrightarrow \frac{1}{b_\infty} = -\frac{\sqrt{2}}{|K|} U(\frac{-1}{2}, 0, \frac{|K|^2}{2b_\infty^2}) \tag{S.8}$$

where U is a confluent hypergeometric function [3]. Equation (S.8) gives the numerical value of b_∞ for a given $|K|$ in the thermodynamic limit.

Linear stability of the complex locked state (S.7) is explored by considering the eigenvalues of the Jacobian matrix

$$\mathbf{J} := \begin{pmatrix} \frac{\partial \dot{x}}{\partial x} & \frac{\partial \dot{x}}{\partial y} \\ \frac{\partial \dot{y}}{\partial x} & \frac{\partial \dot{y}}{\partial y} \end{pmatrix} \Bigg|_{(\mathbf{x}^{(0)\top}, \mathbf{y}^{(0)\top})^\top} \tag{S.9}$$

evaluated at the complex locked state $\boldsymbol{z}^{(0)} = (\mathbf{x}^{(0)^\top}, \mathbf{y}^{(0)^\top})^\top := (x_1^{(0)}, \cdots, x_N^{(0)}, y_1^{(0)}, \cdots, y_N^{(0)})^\top$. Here, we denote $\frac{\partial \dot{x}}{\partial x}$ for a matrix $\left(\frac{\partial \dot{x}_\mu}{\partial x_\nu}\right)_{\mu,\nu \in [N]} \in \mathbb{R}^{N\times N}$ and likewise for others in (S.9). It follows from the Cauchy-Riemann condition of (S.1) that $\mathrm{Eig}(\mathbf{J}) = \mathrm{Eig}(\tilde{\mathbf{J}}) \cup \overline{\mathrm{Eig}(\tilde{\mathbf{J}})}$ where the matrix $\tilde{\mathbf{J}}$ is defined as

$$\begin{aligned} \tilde{J}_{\mu\nu} := \frac{\partial f_\mu}{\partial z_\nu}\bigg|_{\mathbf{z}^{(0)}} &= \mathrm{i}\frac{|K|}{N} \sum_{k=1}^{N} \cos(z_k^{(0)} - z_\mu^{(0)})(\delta_{k\nu} - \delta_{\mu\nu}) \\ &= \mathrm{i}\frac{|K|}{N}\left(\cosh(y_\nu^{(0)} - y_\mu^{(0)}) - \delta_{\mu\nu} \sum_{k=1}^{N} \cosh(y_k^{(0)} - y_\mu^{(0)}) \right) =: \mathrm{i}\frac{|K|}{N} S_{\mu\nu} \end{aligned} \tag{S.10}$$

for $\mu, \nu \in [N]$. Here, $\delta_{\mu\nu}$ is a Kronecker's delta. The matrix $\mathbf{S} := (S_{\mu\nu})_{\mu,\nu\in[N]}$ is real and symmetric, ensuring that it has only zero or real eigenvalues. It then follows that $\mathrm{Eig}(\mathbf{J}) \subset \mathrm{i}\mathbb{R}$. This analysis confirms that the complex locked state (S.7) is linearly neutrally stable for a purely imaginary coupling and for any finite $|K| > 0$. Furthermore, direct numerical integration shows that a trajectory initiated near the complex locked state (S.7) remains close to it, even with an initial perturbation of the order of 10^{-1} away from the complex locked state. Based on this numerical evidence, we conclude that the complex locked state for a purely imaginary coupling is not only linearly neutrally stable but also neutrally stable, given the full nonlinear dynamics.

In the main article, we demonstrate that as $\beta \to 0^+$ the first-order asymptotics well characterizes a complex locked state

$$\begin{aligned} z_\mu^* &= -Q\beta \tanh(y_\mu^{(0)}) + \mathrm{i} y_\mu^{(0)} + \mathcal{O}(\beta^2) \\ &= Q\beta \frac{b\omega_\nu/|K|}{\sqrt{1 + (\frac{b\omega_\mu}{|K|})^2}} - \mathrm{i}\sinh^{-1}\left(\frac{b\omega_\mu}{|K|}\right) + \mathcal{O}(\beta^2) \end{aligned} \tag{S.11}$$

where

$$Q = \frac{\sum_{\nu=1}^{N} \cosh y_\nu^{(0)}}{\sum_{\nu=1}^{N} \cosh y_\nu^{(0)} + \sum_{\nu=1}^{N} \sinh y_\nu^{(0)} \tanh y_\nu^{(0)}} > 0 \tag{S.12}$$

is a positive parameter. This first-order approximation of a complex locked state is further supported by numerical observations, depicted in Fig. S2.

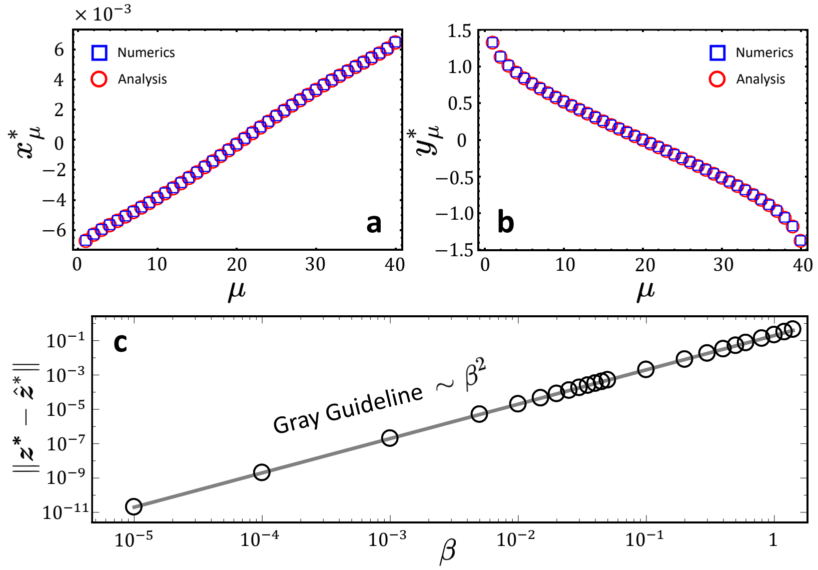

FIG. S2. **Validity of the first-order asymptotic analysis** (a-b) The leading asymptotic behavior of a locked state (S.11) is shown alongside the numerically obtained (exact) results. (c) The norm ($\|\mathbf{z}\|^2 := \langle \mathbf{z}, \mathbf{z} \rangle$) of the difference between the asymptotic estimator $\hat{\boldsymbol{z}}^*$ (S.11) and the actual locked state $\boldsymbol{z}^*$ is depicted on a log-log scale. Parameters are $|K| = 1$ and $N = 40$ in all panels, in panels a) and b), we have $\beta = 0.01$.

SYSTEM SIZE DEPENDENCE OF Q AND W_2

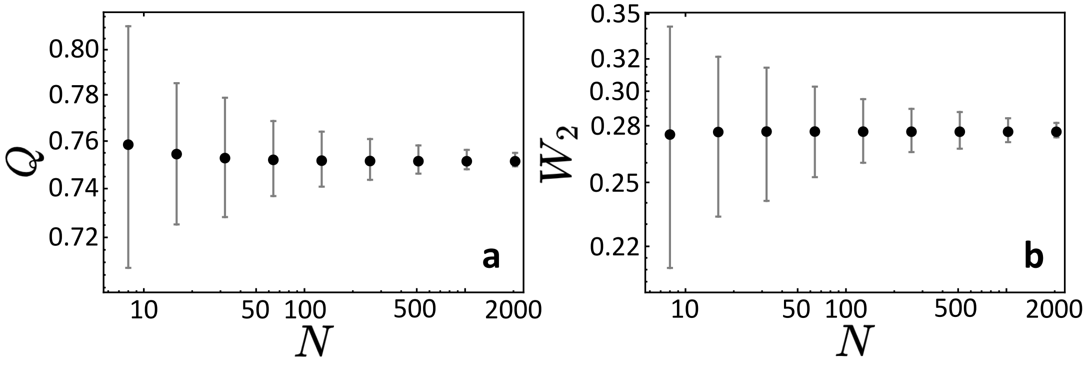

FIG. S3. **System size independence of Q and W_2.** System-size independence of constants Q and W_2. The state-dependent mean-field-like parameters (a) Q and (b) W_2 are shown on a log-log scale from $N = 8$ to $N = 2048$. Black solid dots represent systems with symmetrically drawn natural frequencies, while the error bars indicate the standard deviation of results for 50 different systems with natural frequencies that are generated randomly and independently.

LOCAL ANGLE φ OF SYSTEM STATE VS. COUPLING PARAMETER α

In the main article, we analytically showed that the local angle of a complex locked state asymptotically matches the argument of complex coupling for $N = 2$. Here, we provide a detailed derivation.

Consider a system of two complexified oscillators. The difference $\Delta z := z_2 - z_1$ of the two complex variables under a coupling $K = |K|e^{\mathrm{i}\alpha}$ is governed, after rescaling of time, by

$$\frac{\mathrm{d}}{\mathrm{d}t}\Delta z = c - e^{\mathrm{i}\alpha}\sin\Delta z \tag{S.13}$$

where $c := \frac{|\Delta\omega|}{|K|}$ with $\Delta\omega := \omega_2 - \omega_1$. In Cartesian coordinates with $\Delta z = \Delta x + \mathrm{i}\Delta y$, the governing equation (13) reads

$$\begin{aligned}\frac{\mathrm{d}}{\mathrm{d}t}\Delta x &= c + \sin\alpha\cos\Delta x\sinh\Delta y - \cos\alpha\sin\Delta x\cosh\Delta y\\ \frac{\mathrm{d}}{\mathrm{d}t}\Delta y &= -\cos\alpha\cos\Delta x\sinh\Delta y - \sin\alpha\sin\Delta x\cosh\Delta y\end{aligned} \tag{S.14}$$

in terms of the real and imaginary parts of the state variable $\Delta z = \Delta x + \mathrm{i}\Delta y$.

The two-unit system (S.14) possesses a fixed point

$$\begin{aligned}\Delta x^* &= \sin^{-1}\left(\sqrt{\frac{1+c^2-\sqrt{1+c^4-2c^2\cos 2\alpha}}{2}}\right)\\ \Delta y^* &= -\sinh^{-1}\left(\frac{\sqrt{2}c\sin\alpha}{\sqrt{1-c^2+\sqrt{1+c^4-2c^2\cos 2\alpha}}}\right) < 0\end{aligned} \tag{S.15}$$

for $\alpha \in [0, \frac{\pi}{2}]$. Using (S.15), we analytically obtain the local angle of the complex locked state for $N = 2$ systems

$$\begin{aligned}\tan\varphi := \frac{\Delta y^*}{\Delta x^*} &= \frac{-\sinh^{-1}\left(\frac{\sqrt{2}c\sin\alpha}{\sqrt{1-c^2+\sqrt{1+c^4-2c^2\cos 2\alpha}}}\right)}{\sin^{-1}\left(\sqrt{\frac{1+c^2-\sqrt{1+c^4-2c^2\cos 2\alpha}}{2}}\right)}\\ &= -\tan\alpha + \mathcal{O}(c^2)\end{aligned} \tag{S.16}$$

for any $\alpha \in [0, \frac{\pi}{2}]$ as $c := \frac{|\Delta\omega|}{|K|} \to 0^+$ (or, equivalently, for large coupling strength $|K|$).

LOCAL ANGLES OF A COMPLEX LOCKED STATE

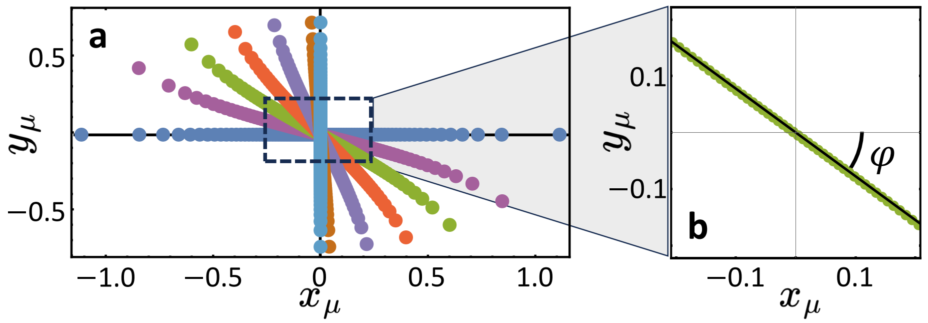

FIG. S4. **Local angle of a complex locked state.** (a) Complex locked states in the complex plane for $N = 80$ and $|K| = 3.0$ move with increasing α values from curves for $\alpha = 0$ to curve for $\alpha = \frac{\pi}{2}$ (see the main text). (b) Local behavior of the complex locked state for $\alpha = 0.6$ (green dots in (a)) is depicted near the origin. The local angle is determined by a slope of a straight line obtained through a linear fit of the data for $x_\mu, y_\mu \in [-0.1, 0.1]$.

DETAILS FOR NUMERICAL INVESTIGATION: FIGURES 1 AND 4

Standard Kuramoto model with unimodal distribution: Fig. 1a

The standard Kuramoto oscillators [4] are described by phase variables $x_\mu \in \mathbb{T} := \mathbb{R}/2\pi\mathbb{Z}$ for $\mu \in [N] := \{1, 2, \cdots, N\}$. They follow

$$\frac{\mathrm{d}}{\mathrm{d}t} x_\mu = \omega_\mu + \frac{K}{N} \sum_{\nu=1}^{N} \sin(x_\nu - x_\mu) \tag{S.17}$$

for $\mu \in [N] := \{1, 2, \cdots, N\}$. The coupling strength $K \in \mathbb{R}_{>0}$ is a real positive parameter and each oscillator is imposed with a constant natural frequency $\omega_\mu \in \mathbb{R}$ drawn from a Cauchy-Lorentz distribution $g(\omega) = \frac{1}{\pi}\frac{1}{1+\omega^2}$ with a constraint $\sum_{\mu=1}^{N} \omega_\mu = 0$. Figure 1a in the main text results from the numerical explorations of Eq. (S.17) with the parameters specified above.

Standard Kuramoto model with bimodal distribution: Fig. 1b

To obtain Fig. 1b, we consider a bimodal distribution

$$g(\omega) = \frac{\Delta}{2\pi}\left(\frac{1}{\Delta^2 + (\omega - \omega_0)^2} + \frac{1}{\Delta^2 + (\omega + \omega_0)^2}\right) \tag{S.18}$$

for $\omega \in \mathbb{R}$. Here, we fix $\Delta = 1/4$. The numerical integration of Eq. (S.17) results in Fig. 1b for each coupling strength K with the condition $\omega_0 = \frac{1.092 \times K}{4}$. For details, see [5].

Van der Pol oscillators: Fig. 4a

We consider an ensemble of Van der Pol oscillators described in [6], which are governed by

$$\begin{aligned}
\frac{\mathrm{d}}{\mathrm{d}t} x_\mu &= y_\mu + K\left(\frac{1}{N}\sum_{\nu=1}^{N} x_\nu - x_\mu\right) \\
\frac{\mathrm{d}}{\mathrm{d}t} y_\mu &= \omega_\mu (1 - x_\mu^2) y_\mu - x_\mu - \varepsilon w(t) \\
\frac{\mathrm{d}}{\mathrm{d}t} w &= -\kappa w - \frac{\varepsilon}{N}\sum_{\nu=1}^{N} x_\nu
\end{aligned} \tag{S.19}$$

for $\mu \in [N]$. The natural frequencies are obtained from $\omega_\mu = 3 - \sigma + \frac{\sigma}{N}(2\mu - 1)$ with $\sigma = 0.15$ for $\mu = 1, 2, \cdots, N$. Here, we vary $K \in \mathbb{R}_{>0}$ as a coupling strength while fixing $\varepsilon = 0.4$ and $\kappa = 1.0$. To measure the order parameter, we define the phase variable for each oscillator as the argument of $x_\mu + \mathrm{i} y_\mu$ for μ.

Stuart-Landau oscillators: Fig. 4b

A system of Stuart-Landau oscillators is governed by

$$\begin{aligned}
\frac{\mathrm{d}}{\mathrm{d}t} \theta_\mu &= \omega_\mu + \frac{K}{N}\sum_{\nu=1}^{N} \frac{r_\nu}{r_\mu} \sin(x_\nu - x_\mu - \alpha) \\
\frac{\mathrm{d}}{\mathrm{d}t} r_\mu &= r_\mu - r_\mu^3 + \frac{K}{N}\sum_{\nu=1}^{N} r_\nu \cos(x_\nu - x_\mu - \alpha)
\end{aligned} \tag{S.20}$$

for $\mu \in [N]$. The natural frequencies are generated from a Gaussian distribution $g(\omega) = \frac{1}{\sqrt{2\pi}} e^{-\omega^2/2\sigma}$ with $\sigma = 0.1$. The phase-lag parameter is fixed during the numerical integration as $\alpha = \frac{\pi}{2} - 0.1$, so $\beta = 0.1$, for each $K \in \mathbb{R}_{>0}$.

EXTREME SYNCHRONIZATION IN A SYSTEM WITH DISORDERED NETWORK TOPOLOGY

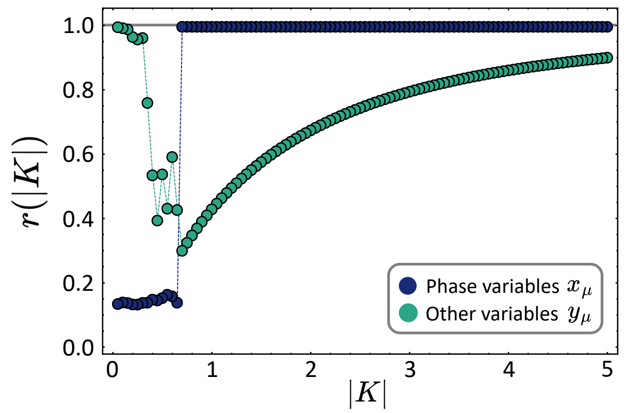

FIG. S5. **Extreme synchronization in a random network.** The order parameter r is depicted as a function of $|K|$ for $N = 40$ and $\alpha = \frac{\pi}{2} - 0.01$: real parts (dark blue) and imaginary parts (green). As the imaginary parts y_ν are unbounded, we define phase-like variables θ_μ by a stereographic projection via $\cos\theta_\mu := \frac{1-y_\mu^2}{1+y_\mu^2}$ and $\sin\theta_\mu := \frac{2y_\mu}{1+y_\mu^2}$ for each μ and evaluate $r = \left|\frac{1}{N}\sum_{\mu=1}^{N} e^{\mathrm{i}\theta_\mu}\right|$. Here, we randomly draw an Erdős–Rényi random network with probability $p = 0.5$ of an edge being present, the largest topological disorder achievable in this ensemble.

EXTREME SYNCHRONIZATION ACROSS DIFFERENT NATURAL FREQUENCY DISTRIBUTIONS

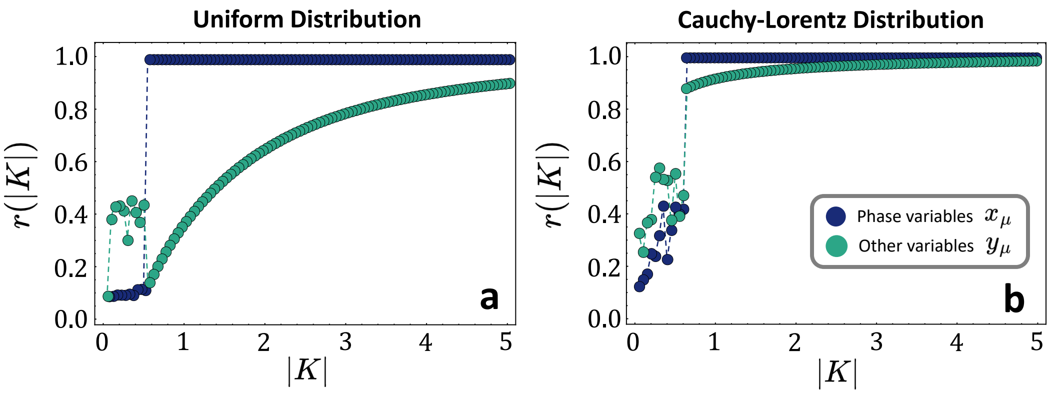

FIG. S6. **Robustness of an extremely discontinuous synchronization transition.** The order parameter r is depicted as a function of $|K|$ for $N = 128$ and $\alpha = \frac{\pi}{2} - 0.01$: real parts (dark blue) and imaginary parts (green): (a) a uniform distribution and (b) a Cauchy-Lorentz distribution.

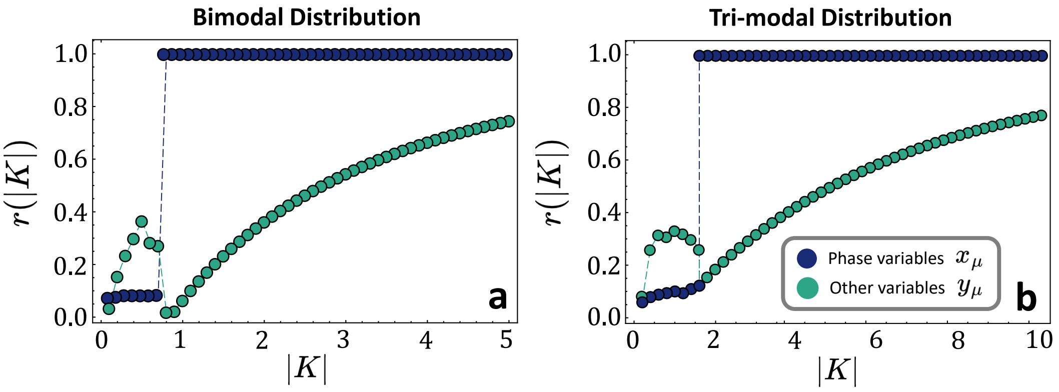

FIG. S7. **Robustness of an extremely discontinuous synchronization transition.** The order parameter r is depicted as a function of $|K|$ for $N = 128$ and $\alpha = \frac{\pi}{2} - 0.01$: real parts (dark blue) and imaginary parts (green): (a) a bimodal Gaussian distribution and (b) a tri-modal Gaussian distribution.

HYSTERESIS AND BASIN SIZE

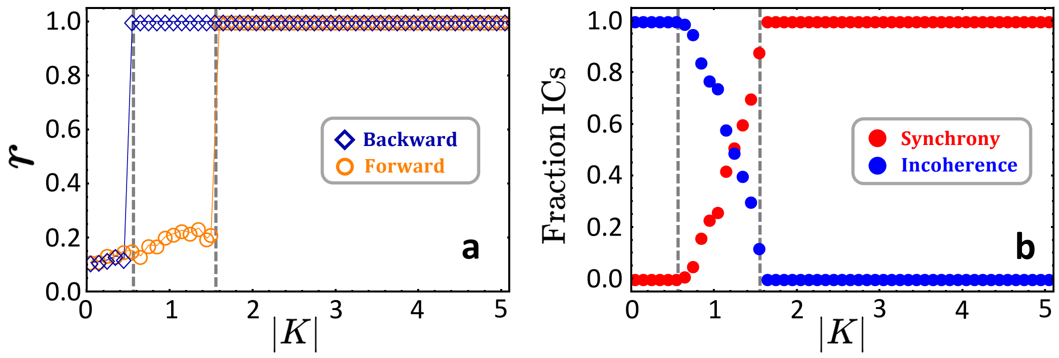

FIG. S8. **Hysteresis loop and basin sizes** (a) The classical Kuramoto order parameter r is depicted as a function of $|K|$ both in the forward (blue diamond) and backward (orange circle) directions. (b) The fractions of random initial conditions drawn independently from a uniform distribution $\mathbb{T} \times [-10^{-3}, 10^{-3}]$ are illustrated, which eventually reach incoherent states (blue) and complexified synchrony (red), respectively with 100 samples. In this figure, the parameters $\beta = 0.01$, $T_{\max} = 4000$ and $N = 40$ are considered.

* seungjae.lee@tu-dresden.de
† marc.timme@tu-dresden.de

[1] M. Thümler, S. G. M. Srinivas, M. Schröder, and M. Timme, Synchrony for Weak Coupling in the Complexified Kuramoto Model, Physical Review Letters **130**, 187201 (2023).

[2] S. Lee, L. Braun, F. Bönisch, M. Schröder, M. Thümler, and M. Timme, Complexified synchrony, Chaos: An Interdisciplinary Journal of Nonlinear Science **34**, 053141 (2024).

[3] S. Hassani, *Mathematical Physics: A Modern Introduction to Its Foundations* (Springer International Publishing, 2013).

[4] S. H. Strogatz, From Kuramoto to Crawford: exploring the onset of synchronization in populations of coupled oscillators, Physica D **143**, 1 (2000).

[5] E. A. Martens, E. Barreto, S. H. Strogatz, E. Ott, P. So, and T. M. Antonsen, Exact results for the Kuramoto model with a bimodal frequency distribution, Physical Review E **79**, 026204 (2009).

[6] G. Ramesan, E. Shajan, and M. D. Shrimali, Explosive synchronization induced by environmental coupling, Physics Letters A **441**, 128147 (2022).
